# Supplementary material for: EnzML: multi-label prediction of enzyme classes using InterPro signatures
Source: BMC Bioinformatics. 2012 Apr 25;13:61. doi: 10.1186/1471-2105-13-61 (PMC3483700; doi:10.1186/1471-2105-13-61)
Supplement: Addtional file 5 — The Java code to format the data files, evaluate and predict. The file enzml_java_code.tar.gz contains the Java code used to format database data to ARFF and XML formats, to execute cross and train-test (jackknife) evaluations and to record evaluation results to database. More information is included in the readme.txt file and the Javadoc files. The code can be used with a MySQL database. To use a different database software, other JDBC drivers might be required. [file 1471-2105-13-61-S5.gz › java_code/utils/doc/uk/ac/ed/inf/utils/class-use/OneToManyMap.html]

Uses of Class uk.ac.ed.inf.utils.OneToManyMap


---


|  |  |  |
| --- | --- | --- |
| **Overview** \| **Package** \| **Class** \| **Use** \| **Tree** \| **Deprecated** \| **Index** \| **Help** | |  |
| PREV   NEXT | **FRAMES**    **NO FRAMES**     **All Classes** |  |


---


## **Uses of Class uk.ac.ed.inf.utils.OneToManyMap**

| Packages that use OneToManyMap | |
| --- | --- |
| **uk.ac.ed.inf.utils** |  |
| **uk.ac.ed.inf.utils.database** |  |
| **uk.ac.ed.inf.utils.test** |  |

Uses of OneToManyMap in uk.ac.ed.inf.utils

| Methods in uk.ac.ed.inf.utils that return OneToManyMap | |
| --- | --- |
| `OneToManyMap` | `TableMap.getMap()`             Get the keys to values map |
| `OneToManyMap` | `TableMap.getReverseMap()`             Get the values to keys map |

| Methods in uk.ac.ed.inf.utils with parameters of type OneToManyMap | |
| --- | --- |
| `void` | `OneToManyMap.addAll(OneToManyMap mapToAdd)`             Add all elements of a one2many map to another map |

Uses of OneToManyMap in uk.ac.ed.inf.utils.database

| Methods in uk.ac.ed.inf.utils.database that return OneToManyMap | |
| --- | --- |
| `OneToManyMap` | `DbReader.getMap(java.lang.String sql, int columnIndexOfKeys, int columnIndexOfValues)`             Converts a query resultset into a map. |

Uses of OneToManyMap in uk.ac.ed.inf.utils.test

| Methods in uk.ac.ed.inf.utils.test that return OneToManyMap | |
| --- | --- |
| `static OneToManyMap` | `OneToManyMapTest.oneToManyMap1()` |
| `static OneToManyMap` | `OneToManyMapTest.oneToManyMap2()` |
| `static OneToManyMap` | `OneToManyMapTest.oneToManyMapEmptyKeyValue()` |

---


|  |  |  |
| --- | --- | --- |
| **Overview** \| **Package** \| **Class** \| **Use** \| **Tree** \| **Deprecated** \| **Index** \| **Help** | |  |
| PREV   NEXT | **FRAMES**    **NO FRAMES**     **All Classes** |  |


---
